# Supplementary material for: Nonalcoholic Fatty Liver Disease Is Related to Abnormal Corrected QT Interval and Left Ventricular Hypertrophy in Chinese Male Steelworkers
Source: Int J Environ Res Public Health. 2022 Nov 6;19(21):14555. doi: 10.3390/ijerph192114555 (PMC9657484; doi:10.3390/ijerph192114555)
Supplement: Supplementary file 1 [file ijerph-19-14555-s001.zip › ijerph-1992728-supplementary.pdf]

# SUPPLEMENTARY DATA

## Non-Alcoholic Fatty Liver Disease is Related to Abnormal Corrected QT Interval and Left Ventricular Hypertrophy in Chinese Male Steelworkers

**Contents**

Measurements of ECG and QT and QTc Interval………………………..2

Echocardiographic Measurements…………………………………………3

## Measurements of ECG and QT and QTc Interval

QT interval was defined as the interval between the first deflection of the QRS complex and the end of the T wave. The end of the T wave was determined by extending a tangent from the steepest portion of the downslope of the T wave until it crossed the T-P segment. The QT and RR intervals were averaged over three consecutive complexes in lead II in sinus rhythm. During other rhythms, QT and RR intervals were averaged over all complexes on the 10-second lead II rhythm strip on the 12-lead ECG. The QTc intervals were calculated using Bazett's formula ($QTc=QT/\sqrt{RR}$) [29,30]. In brief, the ECG tracings were first analyzed by two independent cardiologists and the senior supervising cardiologist who were blinded to the participants' demographics. Inter-reader discrepancies were resolved by direct comparison and adjudicated by the supervising cardiologist. If the T wave amplitude was too flat so that the end of the wave could not be identified or if differences between the QTc measurements between the two independent cardiologist were too great so that they could not be resolved by the supervisor, the data were excluded from the study. The final QTc value was the average of QTc values calculated by the supervisor and the two blinded independent cardiologists. Inter-reader reproducibility assessments for the QT measurements showed a coefficient of reliability of 0.995, and a Pearson's correlation coefficient of 0.995. Comparing inter-reader QT measurements using the paired *t*-test did not achieve

statistical significance (P=0.35). In addition, extremely rapid (>150 bpm) and extremely slow (<40 bpm) heart rate recordings were also excluded to eliminate the influence of heart rate on QT measurements [31,32]. In this study, QTc prolongation was categorized into three sex-specific categories based on the opinion of an ad hoc group according to the latest European regulatory guidelines and a previous study [14].

**Echocardiographic examination**

The echocardiography examinations were performed during a resting period in the morning and in the left lateral position, using a Vivid 7 System (GE Healthcare, General Electric Company, Wauwatosa, USA) with 3-7 MHz transducers and M-mode, two-dimensional and Doppler (pulsed, continuous, color and tissue) echo modalities. Examinations for the long and short parasternal and apical axes, as well as for 2, 3, 4 and 5-chamber views were performed. The cardiac structure and function were assessed using M-mode guided by two-dimensional imaging to obtain the following variables: aortic root end-diastolic diameter; left atrial end-systolic anteroposterior diameter; end diastolic interventricular septal thickness; end diastolic left ventricular posterior wall thickness; left ventricular end-diastolic diameter and volume, and left ventricular end-systolic diameter and volume.

Left atrial (LA) dilatation was defined in the presence of an LA anteroposterior diameter >4.0 cm, and left ventricular (LV) dilatation was defined when the LV diastolic diameter was >5.4 cm. LV ejection fraction was calculated from the apical 4-chamber view using the modified Simpson method. LV mass was calculated using a 2-dimensional method and indexed to the body surface area. Mitral flow was assessed in the apical 4-chamber view using pulsed Doppler. The sample was positioned between the distal extremities of the mitral valve leaflets and then the following variables were obtained: early (E) and late diastolic mitral velocities (A) and E/A ratio.
